# Supplementary material for: Drug Resistance and Molecular Characteristics of Mycobacterium tuberculosis: A Single Center Experience
Source: J Pers Med. 2022 Dec 19;12(12):2088. doi: 10.3390/jpm12122088 (PMC9783070; doi:10.3390/jpm12122088)
Supplement: Supplementary file 1 [file jpm-12-02088-s001.zip › Supplementary Materials-File S1 The process of H&E staining.pdf]

Supplementary Materials

2.6. H&E staining

Cut 1 wax slice of FFPE specimen with 3um thickness. Baked at 72 degrees for 30 min, xylene for 10 min × 2 times, gradient ethanol for 5 min × 3 times, hematoxylin solution for 2 min, hydrochloric acid alcohol fractionation for 5 s, ammonia for 5 s, eosin Y solution for 10 s. After rapid dehydration in gradient ethanol and transparency in xylene for 3 times, the slice was sealed with neutral resin.
