# Supplementary figures and images for: Drug Resistance and Molecular Characteristics of Mycobacterium tuberculosis: A Single Center Experience
Source: J Pers Med. 2022 Dec 19;12(12):2088. doi: 10.3390/jpm12122088 (PMC9783070; doi:10.3390/jpm12122088)

Supplementary Materials

Figure S1. some mutation types on membrane strips.

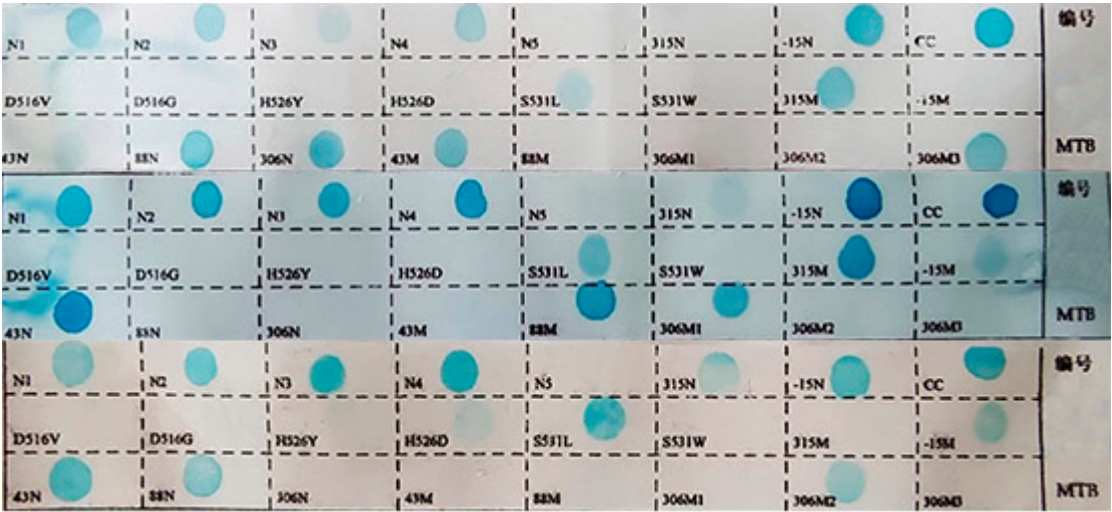

Supplement: Supplementary file 1 [file jpm-12-02088-s001.zip › Supplementary Materials-Figure S1. some mutation types on membrane strips.pdf]
